# Prescribing Trend of Antirheumatic Drugs in Taiwan and Risk of Cardiovascular Disease in Patients with Rheumatoid Arthritis: A Nationwide Cohort Study

**DOI:** 10.1155/2019/7987529

**Published:** 2019-02-20

**Authors:** Chien-Ying Lee, Chih-Jaan Tai, Ya-Fang Tsai, Yu-Hsiang Kuan, Chiu-Hsiang Lee, Kuang-Hua Huang

**Affiliations:** ^1^Department of Pharmacology, Chung Shan Medical University, Taichung 40242, Taiwan; ^2^Department of Pharmacy, Chung Shan Medical University Hospital, Taichung 40242, Taiwan; ^3^Department of Health Services Administration, China Medical University, Taichung 40402, Taiwan; ^4^Department of Otorhinolaryngology, China Medical University Hospital, Taichung 40402, Taiwan; ^5^Department of Health Policy and Management, Chung Shan Medical University, Taichung 40242, Taiwan; ^6^Institute of Medicine, Chung Shan Medical University, Taichung 40242, Taiwan; ^7^Department of Nursing, Chung Shan Medical University Hospital, Taichung 40242, Taiwan

## Abstract

We aimed to investigate the prescribing trend of antirheumatic drugs and assess the risk of cardiovascular disease in patients with rheumatoid arthritis in Taiwan. This study was a retrospective cohort study, conducted based on the Taiwan National Health Insurance Research Database. The study subjects were 15,366 new rheumatoid arthritis patients from 2003 to 2010. To avoid selection bias, we applied propensity score matching to obtain general patients, as the control group. Cox proportional hazard model was used to evaluate the risk of cardiovascular disease in rheumatoid arthritis patients. The most common prescriptions of rheumatoid arthritis were nonsteroidal anti-inflammatory drugs. After controlling for related variables, rheumatoid arthritis patients had a higher risk of cardiovascular disease than general patients (adjusted hazard ratio [aHR] = 1.31; 95% confidence interval [CI]: 1.23-1.39). Age was the most significantly associated risk factor with the cardiovascular disease. Other observed risk factors for cardiovascular disease included hypertension (aHR = 1.57, 95% CI: 1.48-1.65), diabetes mellitus (aHR = 1.47, 95% CI: 1.38-1.57), and chronic kidney disease (aHR = 1.48, 95% CI: 1.31-1.66). Patients with rheumatoid arthritis indeed had a higher risk of incident cardiovascular diseases. Besides, age, hypertension, diabetes mellitus, and chronic kidney disease were also associated with a higher risk of cardiovascular disease.

## 1. Introduction

Patients with rheumatoid arthritis (RA) have 2-5 times increased the risk of premature cardiovascular disease (CVD), which shortens their life expectancy by 5-10 years [1]. Many studies have emerged between the paradigm of inflammation in the pathogenesis of atherosclerosis and the well-established mechanisms of inflammation in the pathogenesis of RA [1, 2]. Common proinflammatory cytokines are involved in the development and progression of both RA and atherosclerosis. Proinflammatory cytokines, such as interleukin- (IL-) 1, IL-6, and tumor necrosis factor-alpha (TNF-*α*), produced within locally affected joints in RA may promote both traditional [e.g., dyslipidemia and insulin resistance (IR)] and nontraditional (e.g., oxidative stress) systemic cardiovascular risk factors [2].

A study reported that RA patients have a significantly higher risk of coronary heart disease than do non-RA patients. RA patients are less likely to report symptoms of angina and more likely to experience unrecognized myocardial infarction (MI) and sudden cardiac death [3]. Another population-based study indicated that congestive heart failure (CHF) incidence rates were 1.99 and 1.16 cases per 100 person-years in RA and non-RA patients, respectively [4].

Because inflammation plays crucial roles in RA and CVD as well as in the increased cardiovascular risk in RA, the suppression of inflammation can ultimately lower the cardiovascular morbidity and mortality in RA patients [5]. Antirheumatic drugs include glucocorticosteroids, nonsteroidal anti-inflammatory drugs (NSAIDs), disease-modifying antirheumatic drugs (DMARDs), and biological agents (e.g., anti-TNF-*α* therapy). In inflammatory disorders, the suppression of systemic inflammation favoring atherosclerosis may lead to an improvement in cardiovascular prognosis. In RA, the attenuation of inflammatory joint disease through the administration of anti-TNF-*α* therapy, or probably any powerful disease-modifying antirheumatic drug, is associated with a concomitant reduction in the risk of cardiovascular events [6].

Patients with RA may have a higher risk of CVD; understanding the relationship between these conditions is important for clinicians. This retrospective cohort study was conducted to investigate the prescribing trend of antirheumatic drugs and assess the risk of CVD in patients with RA by using nationwide data from the National Health Insurance Research Database (NHIRD) in Taiwan.

## 2. Materials and Methods

### 2.1. Data Source

This nationwide retrospective cohort study was based on the data from Longitudinal Health Insurance Database (LHID), which was randomly selected from the NHIRD provided by the National Health Insurance Administration, Ministry of Health and Welfare, and managed by National Health Research Institutes (Registered number NHIRD-104-004). The LHID comprises the data of detailed clinical records from patient visits, primary and secondary diagnostic codes, and prescription orders for one million randomly selected beneficiaries of the National Health Insurance (NHI) program. The NHI program of Taiwan has been launched since 1995 and >99% of citizens are all enrolled in the program. Therefore, the NHIRD can represent the utilization conditions of medical resources for the 23 million residents and is one of the largest databases universally. This study was exempted from informed consent because the personal identification data were encrypted and transformed in the NHIRD. This study protocol was approved by the Institutional Review Board of China Medical University and Hospital, Taiwan (IRB No.: CMUH103-REC-002).

### 2.2. Study Subjects

The case group in the study was patients with new diagnosis RA. The definition of RA patients was RA diagnosis three times a year, according to the International Classification of Diseases, Ninth Revision, Clinical Modification (ICD-9-CM) code 714 and receiving at least one of these drugs (i.e., steroids, NSAIDs, or DMARDs). We excluded patients who received CVD diagnosis before RA to reduce the study bias. Furthermore, we used the propensity score matching (PSM) to obtain general patients, in 1:5 matching for each RA patient, as the control group. The general patients who we selected for the control group had not received any RA diagnosis during the study period. The propensity score is the probability that a unit with certain characteristics will be assigned to RA patients. The scores could be used to reduce or eliminate selection bias in observational studies by the characteristics of RA patients and general patients. The characteristics we selected for matching were gender, age, premium-based monthly income, and urbanization.

### 2.3. Study Designs

**Figure 1:**
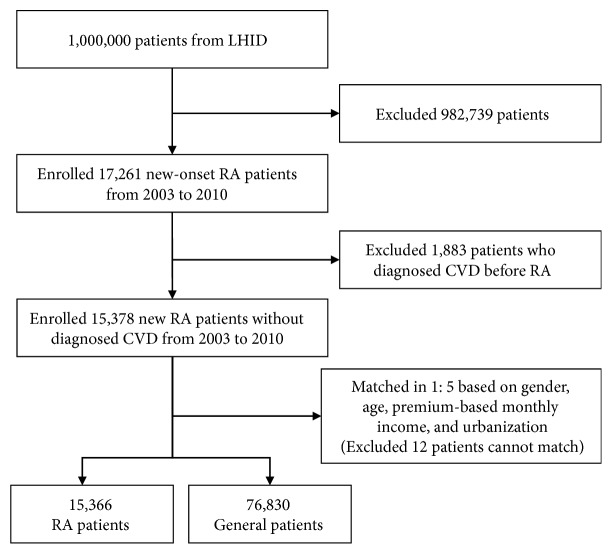
Flowchart of the study subject selection process.

This study was conducted using 2002-2013 claim data from LHID. We enrolled RA patients and general patients from 2003-2010 as the study subject, to ensure that exclusion condition and each patient had at least 3 years of follow-up.  Figure 1 lists flowchart of selection patients for inclusion. We enrolled 15,366 RA patients and 76,830 general patients between 2003 and 2010. Each patient was follow-up until the date of incident CVD, death, or the end of 2013, whichever occurred first. We calculated the prescriptions of antirheumatic drugs during the study period and estimated the risk of CVD in RA patients. The definition of CVD in the study contained acute myocardial infarction (MI) (ICD-9-CM code 410), angina pectoris (ICD-9-CM code 413), congestive heart failure (CHF) (ICD-9-CM code 428.0), left heart failure (ICD-9-CM code 428.1), and heart failure, unspecified (ICD-9-CM code 428.9).

The study used the Cox proportional hazard model to estimate the hazard ratios with 95% confidence interval (CI) for the association between RA and CVD, after controlling for related variables. Control variables were sex, age, and comorbidity diseases. The comorbidity disease contained hypertension (HTN) (ICD-9-CM codes 401-405), hyperlipidemia (HPL) (ICD-9-CM code 272), metabolic syndrome (MS) (ICD-9-CM code 277), diabetes mellitus (DM) (ICD-9-CM code 250), and chronic kidney disease (CKD) (ICD-9-CM codes 580-588).

All statistical analyses in the study were using SAS software version 9.4 (SAS Institute Inc., Cary, NC, USA) and SPSS version 18 (IBM SPSS Inc., Chicago, IL, USA). Statistical significance in this study was defined as p < 0.05.

## 3. Results

### 3.1. The Characteristic Distribution of Study Subject after Matching

Table 1 lists the basic characteristics of RA patients and general patients after matching. A total of 92,196 patients aged >34 years were selected from a nationally representative sample of Taiwan's LHID from 2003 to 2010. Among them, 15,366 and 76,830 were RA patients and general patients, respectively; 68.09% were women and 31.91% were men. The average age of RA patients was 50.01 years and standard deviation (SD) was 16.02 years; the age of general patients was 49.26 ± 17.07 years. As expected, the characteristics distribution of RA and general patients was no significant differences after matching.

### 3.2. The Prescribing Trend of Antirheumatic Drugs

**Figure 2:**
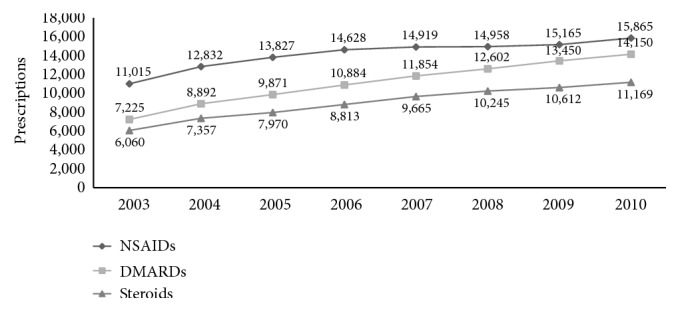
The prescribing trend of antirheumatic drugs from 2003 to 2010.

Figure 2 showed the prescribing trend of antirheumatic drugs from 2003 to 2010. NSAIDs were the most commonly used drug since 2003 (11,015 prescriptions in 2003 to 15,865 prescriptions in 2010). In addition, DMARDs increased over two times from 2003 to 2010 (7,225 prescriptions in 2003 and 14,150 prescriptions in 2010). Steroids were only 6,060 prescriptions in 2003 and rose to 11,169 prescriptions in 2010.

### 3.3. The Risk of CVD in RA Patients

**Figure 3:**
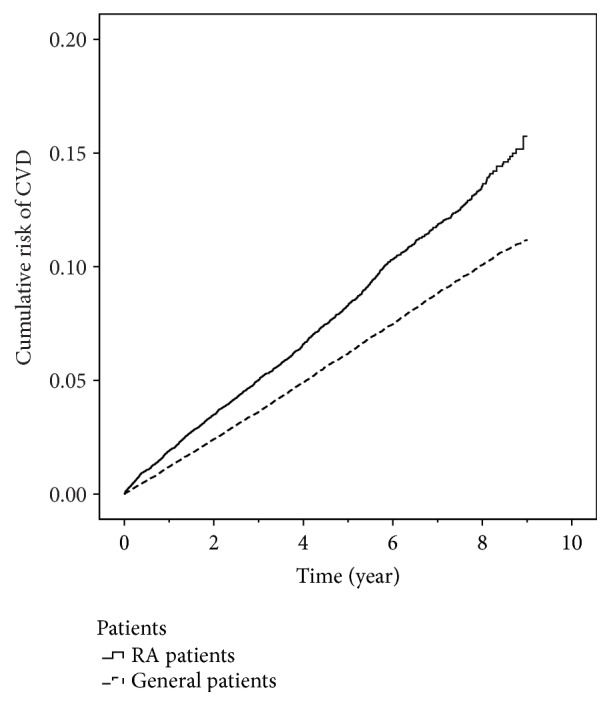
The cumulative risk of CVD between RA patients and general patients (p-value < 0.001, Log-rank test).

As Table 2, among the 92,196 participants, 6,665 patients (7.23%) had CVD. A higher percentage of RA patients had CVD than general patients (8.56% versus 6.96%). The cumulative risk of CVD was significantly higher in RA patients than in general patients (Figure 3; Log-rank test, p-value < 0.001). The age of patients with CVD was 64.85 ± 12.43 years; the age of patients without CVD was 48.19 ± 16.60 years. The patients with HTN had a higher percentage on incident CVD, compared with the patients without HTN (13.18% versus 6.60%). The patients with other comorbidity disease also had a higher percentage on incident CVD, compared with the patients without comorbidity disease (HPL: 14.79% versus 7.20%; MS: 10.22% versus 7.22%; DM: 18.01% versus 6.33%; CKD: 18.84% versus 7.03%). In addition, the distribution of the most covariates associated with CVD was statistically significant difference, except MS.

Table 3 shows the hazard ratios (HR) for CVD among RA patients and general patients. The average follow-up time was 5.04 years in RA patients and 5.60 years in general patients, respectively. After controlling for other relevant influencing factors, RA patients had a higher risk being adjusted HR of 1.31 (95% CI: 1.23-1.39) than general patients. Compared with patients without HTN, the adjusted HR was 1.57 (95% CI: 1.48-1.65) in those with HTN. Compared with patients without DM, the adjusted HR was 1.47 (95% CI: 1.38-1.57) in those with DM. Compared with patients without CKD, the adjusted HR was 1.48 (95% CI: 1.31-1.66) in those with CKD. Furthermore, the age was the greatest risk factors contributing to the occurrence of incident CVD. The risk of CVD was higher in elderly patients than in those aged ≦34 years; the adjusted HR was 17.65 (95% CI: 13.97-22.31) in 55-64-year-old patients and 40.32 (95% CI: 31.99-50.84) in ≥65-year-old patients.

## 4. Discussion

The study results demonstrated that RA patients had a higher risk of CVD than general patients. Patients with comorbidities contained HTN, DM, and CKD increased the risk of CVD. In addition, study results also provided evidence that elderly patients were at a higher risk of CVD than the patients aged less than 34 years.

RA is associated with an increased burden of CVD, which is at least partially attributed to classical risk factors such as HTN and dyslipidemia [7, 8]. HTN is one of the most important modifiable risk factors for CVD in the general population [9] and is more common in patients with RA. HTN in patients with RA is associated with increasing concentrations of homocysteine and leptin. The pathogenesis of HTN in RA may involve pathways more regularly associated with fat and vascular homeostasis [10]. RA patients with higher erythrocyte sedimentation rates and the presence of extra-articular manifestations were more likely to exhibit dyslipidemia [11]. The prevalence of dyslipidemia in Japanese patients with RA is higher than that in the non-RA population. Controlling RA disease activity might improve lipid profiles and lower CVD risk. A low dose of atorvastatin was effective for the treatment of dyslipidemia in RA patients but had no apparent effect on RA disease activity [12]. In our study, HTN was a risk factor for CVD in patients with RA. This result is similar to that of other studies.

Patients with RA have characteristics that indicate a high risk of type 2 DM. Controlling inflammation may improve insulin sensitivity and subsequently reduce the risk of type 2 DM in RA patients. This may also reduce the risk of CVD in high-risk groups [13]. One study indicated that RA is apparently associated with an increased risk of type 2 DM in Taiwan. Compared with non-RA, the OR of RA for type 2 DM was 1.68 (95% CI: 1.53-1.84) in men and 1.46 (95% CI: 1.39-1.54) in women [14]. In our study, DM was identified as a risk factor for CVD, which is similar to the results of previous studies. A systematic review and meta-analysis indicated that HTN, type 2 DM, smoking, hypercholesterolemia, and obesity increased CVD risk in patients with RA [15].

Renal disease in RA is clinically important. The previous study demonstrated that RA patients are more prone to have CKD. This could have serious implications, as the majority of RA patients use NSAIDs and different immunosuppressive drugs [16]. Another study indicated that the strong association between mild CKD and CVD has been shown and reported that end-stage renal disease (ESRD) is strongly associated with an increase in CVD mortality [17, 18]. In our study, CKD was a risk factor for CVD in patients with RA, which is consistent with the results of previous studies.

We also identified that the risk of CVD was higher in elderly patients with RA. Accelerated aging caused by the senescence of multiple systems represents an attractive biological model that may partly explain the increase in CVD and mortality rates observed in RA [19]. One study indicated that age exerts an exponentially increasing effect on CVD risk in seropositive RA but no increased effect among seronegative patients. The effect of age on CVD risk in seropositive RA was nearly double its effect on the general population, with additional log (age) coefficients of 2.91 for women (p = 0.002) and 2.06 for men (p = 0.027) [20]. This finding is similar to that of our study.

Antirheumatic drugs include glucocorticosteroids, NSAIDs, DMARDs, and biological agents. NSAIDs and oral glucocorticosteroids likely contribute to the increased CVD risk in RA [21]. A recent meta-analysis suggested that all NSAIDs confer some CVD risk. The CVD risks or benefits associated with the use of DMARDs and/or biological agents as well as the role of statins in RA remain controversial [22].

As the study results, the proportion of NSAID prescriptions was highest from 2003 to 2010 in Taiwan. Nonselective NSAIDs and coxibs are commonly used in RA, and their effects on renal function and blood pressure should be considered, especially in patients with preexisting HTN or renal impairment and in elderly patients with the most pronounced side effects [23]. One study revealed that MI in RA is associated with demographic and cardiovascular risk factors and corticosteroid use. The covariate-adjusted risk of first MI in RA versus that in noninflammatory rheumatic disorders was 1.9 (95% CI: 1.2-2.9, p = 0.005). The use of corticosteroids was associated with the future development of DM and HTN [24]. Patients treated with high-dose steroids (>7.5 mg/day of prednisone) appear to have twice the risk of heart disease compared with those not treated with steroids [25].

Because of their robust disease-modifying effects on both proinflammatory and proatherogenic pathways, biological treatments have been substantially speculated to serve as a highly effective RA disease control [26]. Methotrexate (MTX) is considered the first-line DMARD agent for RA. One study reported that MTX use is associated with a reduced risk of CVD events in patients with RA. In RA, the reduction of inflammation through MTX use not only improves disease-specific outcomes but may also reduce collateral damage such as atherosclerosis [27]. Rheumatoid factor positivity and erosions both increased CVD risk, with ORs of 2.04 (1.02-4.07) and 2.36 (0.92-6.08), respectively. MTX and, to a lesser extent, sulfasalazine were associated with a significantly lower CVD risk than that in RA patients who never used sulfasalazine, hydroxychloroquine, or MTX. DMARD use, in particular MTX use, results in the powerful suppression of inflammation, thereby reducing the development of atherosclerosis and subsequently clinically overt CVD [28].

Anti-TNF-*α* therapy is associated with a significantly increased risk of HTN in patients with RA. Physicians should be aware of this risk and provide continual monitoring in patients receiving these therapies [29]. Type 2 DM is associated with IR, which is partially caused by TNF-*α*, a major effecter of IR in humans with type 2 DM. Anti-TNF-*α* therapy for autoimmune diseases resulted in a significant improvement in the fasting blood glucose, glycated hemoglobin, and triglyceride values of such patients [30]. In an inception RA cohort, anti-TNF-*α* therapy was associated with a 51% reduction in the risk of DM [31]. One study reported that, among patients with RA, the multivariate adjusted ORs for DM were 0.62 (95% CI = 0.42-0.91) for anti-TNF-*α* therapy, 0.77 (95% CI = 0.53-1.13) for MTX, and 0.54 (95% CI = 0.36-0.80) for hydroxychloroquine compared with other nonbiological DMARDs [32].

Strengths of the study are that it was based on a large and representative population cohort, extracted from the NHI system covering 99% population in Taiwan, avoiding bias from selection, nonresponse, or poor recall. The NHIRD has been shown to have good levels of accuracy and completeness in recording prescriptions and clinical diagnoses. Besides, we not only adjusted for many potential confounding factors but also used the propensity score matching to select general patients as the control group. Therefore, the study indicates that RA patients increase of 1.31 times the risk of CVD, with a narrower and statistically significant confidence interval.

There are a few limitations in the study. NHRID do not contain patients' biochemical test data. However, in order to avoid miscoding problems, we use the ICD-9-CM code of RA and antirheumatic drugs to select RA patients to improve diagnostic accuracy. In addition, some factors affecting the CVD cannot be obtained from the NHI database. For example, life-related variables from these patients cannot be included in the analysis, such as BMI, alcohol consumption, and tobacco consumption behavior. Moreover, the medications, such as NSAIDs and corticosteroids, may increase the risk of CVD. The medication is the confounding factor really extremely to control, especially in the retrospective cohort study. Each patient in the study period may receive inconsistent prescription patterns in the follow-up period, including drug type, drug dose, and medication duration. Therefore, our study reduced the medication confounding by adjusted comorbidity disease. This study was a nationwide population-based study. Thus, the study results have the accuracy and representativeness, although without the medication status. However, this study can only provide evidence to prove the association between RA and CVD, which cannot represent the cause-effect relation. It is necessary to obtain this information from other databases or questionnaires to analyze its influence on their relationship in future research.

## 5. Conclusions

The proportion of NSAID prescriptions was highest in Taiwan. The study highlights the significant increase in the risk of developing CVD in patients with RA. The study further corroborated this relationship via the Cox proportional hazard model. HTN, DM, and CKD were related to incident CVD. Furthermore, age is the greatest risk factors contributing to the occurrence of incident CVD.

## Figures and Tables

**Table 1:** The characteristics of RA and general patients (matched in 1:5 scales).

Variables	General patients		RA patients		Total		p-value ^1^
	N	%	N	%	N	%	
Total	76,830	100	15,366	100	92,196	100	
Gender							>0.99
Male	24,515	319.91	4,903	31.91	29,418	31.91	
Female	52,315	68.09	10,463	68.09	62,778	68.09	
Age (year)	49.26 ± 17.07		50.01 ± 16.02		49.39 ± 16.90		>0.99
≦34	12,985	16.9	2,597	16.9	15,582	16.9	
35-44	13,400	17.44	2,680	17.44	16,080	17.44	
45-54	20,860	27.15	4,172	27.15	25,032	27.15	
55-64	14,698	19.13	2,939	19.13	17,637	19.13	
≧65	14,887	19.38	2,978	19.38	17,865	19.38	
Premium-based Monthly income (NTD)							>0.99
Insured dependent	20,015	26.05	4,003	26.05	24018	26.05	
<17,280	12,865	16.74	2,573	16.74	15438	16.74	
17,281~22,800	23,763	30.93	4,752	30.93	28515	30.93	
≧22,801	20,187	26.27	4,038	26.27	24225	26.27	
Urbanization							>0.99
Level 1	22,753	29.61	4,550	29.61	27,303	29.61	
Level 2	24,450	31.82	4,890	31.82	29,340	31.82	
Level 3	12,156	15.82	2,431	15.82	14,587	15.82	
Level 4	10,655	13.87	2,131	13.87	12,786	13.87	
Level 5	1,377	1.79	276	1.79	1,653	1.79	
Level 6	2,605	3.39	521	3.39	3,126	3.39	
Level 7	2,834	3.69	567	3.69	3,401	3.69	

^1^Used Chi-square test to exam the characteristics distribution.

**Table 2:** Covariates associated with CVD with univariate analysis.

Variable	Without CVD		With CVD		Total		p-value ^1^
	N	%	N	%	N	%	
Total	85,531	100	6,665	100	92,196	100.00	
Patients							<0.001
General	71,480	93.04	5,350	6.96	76,830	83.33	
RA	14,051	91.44	1315	8.56	15,366	16.67	
Gender							<0.001
Male	26,996	91.77	2,422	8.23	29,418	31.91	
Female	58,535	93.24	4,243	6.76	62,778	68.09	
Age (year)	48.19 ± 16.60		64.85 ± 12.43				<0.001
≦34	15,508	99.53	74	0.47	15,582	16.9	
35-44	15,806	98.30	274	1.70	16,080	17.44	
45-54	23,996	95.86	1036	4.14	25,032	27.15	
55-64	16,076	91.15	1561	8.85	17,637	19.13	
≧65	14,145	79.18	3720	20.82	17,865	19.38	
HTN							<0.001
No	77,886	93.40	5,504	6.60	83,390	90.45	
Yes	7,645	86.82	1,161	13.18	8,806	9.55	
HPL							<0.001
No	85,220	92.80	6,611	7.20	91,831	99.6	
Yes	311	85.21	54	14.79	365	0.4	
MS							0.176
No	85,408	92.78	6,651	7.22	92,059	99.85	
Yes	123	89.78	14	10.22	137	0.15	
DM							<0.001
No	79,705	93.67	5,385	6.33	85,090	92.29	
Yes	5,826	81.99	1280	18.01	7,106	7.71	
CKD							<0.001
No	84,243	92.97	6,366	7.03	90,609	98.28	
Yes	1,288	81.16	299	18.84	1,587	1.72	

^1^Used Chi-square test to exam the characteristics distribution.

^2^RA, rheumatoid arthritis; HTN, hypertension; HPL, hyperlipidemia; MS, metabolic syndrome; DM, diabetes mellitus; CKD, chronic kidney disease.

**Table 3:** Risk of CVD in RA patients with multivariable analysis of Cox regression analysis.

Variables	Unadjusted	95% CI	p-value	Adjusted	95% CI	p-value
	HR			HR		
Patients						
General (ref.)	1			1		
RA	1.36	1.28-1.45	<0.001	1.31	1.23-1.39	<0.001
Gender						
Male (ref.)	1			1		
Female	0.83	0.79-0.87	<0.001	0.82	0.78-0.86	<0.001
Age (year)						
≦34 (ref.)	1			1		
35-44	3.56	2.75-4.60	<0.001	3.50	2.71-4.53	<0.001
45-54	9.11	7.20-11.54	<0.001	8.56	6.76-10.83	<0.001
55-64	20.62	16.33-26.03	<0.001	17.65	13.97-22.31	<0.001
≧65	52.41	41.64-65.97	<0.001	40.32	31.99-50.84	<0.001
HTN						
No (ref.)	1			1		
Yes	3.75	3.57-3.94	<0.001	1.57	1.48-1.65	<0.001
HPL						
No (ref.)	1			1		
Yes	2.29	2.15-2.44	<0.001	0.99	0.93-1.07	0.963
MS						
No (ref.)	1			1		
Yes	1.58	0.94-2.67	0.086	0.91	0.54-1.53	0.717
DM						
No (ref.)	1			1		
Yes	3.24	3.04-3.44	<0.001	1.47	1.38-1.57	<0.001
CKD						
No (ref.)	1			1		
Yes	2.93	2.61-3.29	<0.001	1.48	1.31-1.66	<0.001

RA, rheumatoid arthritis; HTN, hypertension; HPL, hyperlipidemia; MS, metabolic syndrome; DM, diabetes mellitus; CKD, chronic kidney disease.

## Data Availability

The National Health Insurance Research Database (NHIRD) used to support the findings of this study was provided by National Health Research Institutes under license and so cannot be made freely available. Requests for access to these data should be made to National Health Research Institutes (https://nhird.nhri.org.tw).
